# Biosurfactants as an alternative eco-friendly solution for water-in-diesel emulsions-A review paper

**DOI:** 10.1016/j.heliyon.2024.e37485

**Published:** 2024-09-06

**Authors:** Asghar Ali, A. Rashid A. Aziz, Mhadi A. Ismael, Saeed Alqaed

**Affiliations:** a Center of Sustainable Resources for Intelligent and Efficient Mobility (CSRIEM), Mechanical Engineering Department, Universiti Teknologi PETRONAS, Seri Iskandar, Perak, 32610,Malaysia; b Mechanical Engineering Department, MUET SZAB Campus Khairpur Mir's, 66020, Pakistan; c Engineering Department, College of Engineering, Najran University, Najran, 61441, Saudi Arabia

**Keywords:** WIDE, Emulsion, Surfactant, Biosurfactant, Emulsified fuel properties, Micro-explosion

## Abstract

Diesel engines are used extensively in heavy-duty transportation due to their high thermal efficiency and energy density, but they also contribute to environmental pollution. Water-in-diesel emulsions have emerged as an alternative method for decreasing NOx and emissions, but there are still obstacles to assuring engine performance and stability. Surfactants are used to stabilise the emulsion by decreasing the interfacial tension between the fuel and water. Studies on water-in-diesel emulsions published literature suggest that chemical surfactants have been used in the production of emulsified fuels. In addition, research have shown that biosurfactants are less harmful to the environment than chemical surfactants. However, only limited study has been conducted on the use of biosurfactants in emulsified fuel. Consequently, it is important to investigate the possible use of biosurfactants in applications using emulsified fuels. This research studies the categorization of surfactants and biosurfactants and emulsion methods for the development of emulsified fuel. This research also aids in the selection of the most suitable surfactant and biosurfactant for applications, particularly in the context of water-in-diesel emulsions and diesel-in-water emulsions, with the goal of developing an environmentally friendly, stable emulsified fuel that can reduce the emission effect and protect the environment.

## 1 Introduction

**Fig. 1:**
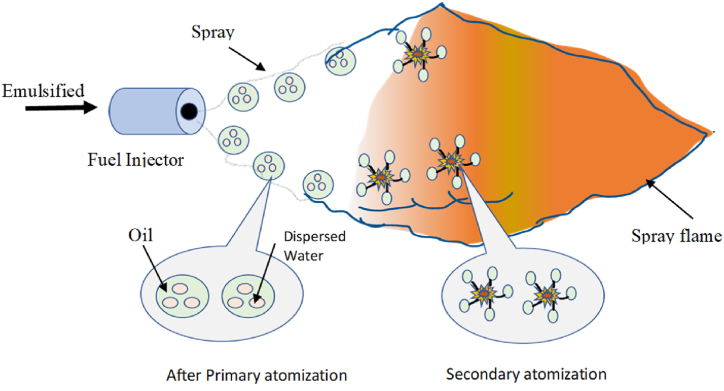
Illustrate the micro-explosion process [13].

Diesel engine is one of the key sources of energy for ships, factories, and automobiles. Unfortunately, the Widespread use of diesel engines in the modern era creates environmental hazards that affect all living species in the universe. Several studies have been conducted in recent years to determine ways to cut down on the emission of harmful gases from diesel engines. To do so, diesel is emulsified with the right amount of water to lower pollution like carbon monoxide and nitrogen oxides (NOx). Additionally, emulsion fuel technology improves diesel engine thermal efficiency [1]. WIDE (Water-in-Diesel Emulsion) is a good alternative fuel that cuts down on harmful pollutants and improves combustion efficiency [1]. All previous studies suggested that adding water into diesel fuel for CI engines contributes to emission reductions. because it improves combustion efficiency due to secondary atomization, thus reducing NOx emissions and fuel consumption. Furthermore, when WIDE is heated to high temperatures, dissociation may occur, resulting in the creation of hydroxyl radicals, which aid in the oxidation of soot and thereby reduce soot emissions [2]. The diesel engine is one of the most significant sources of CO2. Water injection into the combustion chamber has become very popular to minimise emissions from a diesel engine. According to Canfield, water injection causes an increase in the ignition delay before combustion, which has an impact on the combustion process. This technique will aid in the reduction of pollutants, primarily nitrogen oxide (NOx), as well as the synthesis of carbon dioxide (CO2) [3]. As indicated in the literature, most studies have demonstrated that substituting water-in-diesel fuel for diesel fuel greatly reduces emissions while somewhat lowering engine performance when using clean diesel fuel. Moreover, WIDE also helps reduce emissions [4]. Other studies were conducted to investigate how well a diesel engine performed when utilising WIDE as fuel and adding hydrogen (H2). The H2 mixture injection in the combustion engine was found to be effective. (hydrogen volume utilised: 0 %, 0.6 %, and 1.2 %). This will lead to improved thermal efficiency but reduced break-specific fuel consumption [5]. Furthermore, the stability of WIDE fuel was investigated. Since surfactants help to reduce the surface tension of diesel and water molecules, they are employed to stabilise emulsion fuel. Surfactants consist of a hydrophilic head (polar) and a hydrophilic tail (non-polar). The polar group in a water-diesel emulsion turns toward water, while the non-polar group turns toward diesel, reducing the interfacial tension (IFT) between the two phases [6]. The experimental examination was conducted to determine the influence of surfactant addition on the preparation of WIDE. In that study, a significant amount of water was added to the test fuel. 5–15 percent by weight, diesel (94–84 percent by weight), and surfactant (1 % by weight). The addition of a 1 % surfactant resulted in simultaneous reductions of NOx, percent w/w CO, and percent w/w HC. At low water concentrations, the surfactant mixture greatly reduced NOx emissions [7]. On the other hand, the micro-explosion phenomena of WIDE and Its influencing parameters have a significant impact on CI engine combustion. This article explains the basics of micro-explosions and the factors that influence them. The micro-explosion of emulsion droplets has a significant impact on atomization, combustion efficiency, and pollutant emissions [[8], [9], [10]]. In WIDE, water is dispersed in the droplets of diesel due to the use of surfactant. When the emulsified fuel is sprayed on a hot surface, the surface of the droplets starts heating [11]. Because the boiling point of oil is typically greater than that of water, water droplets inside oil can be “superheated,” or heated to a higher temperature than its normal boiling point. This physical condition is called a “metastable one.” The water begins to rapidly boil when there is an interruption. In an instant, the parent oil droplet is split by explosive boiling. Fig. 1 illustrate the phenomena of micro-explosion [12].

When emulsion droplets are exposed to higher temperatures, they go through a process called “preferential evaporation,” which causes “puffing” and “micro-explosions” that break up the droplets into smaller pieces that help the mixture mix better [14]. It was found that WIDE had a spontaneous explosion during combustion [15]. This made it easy for the smaller fuel particles to get in touch with the air and burn completely. This cut down on the production of PM, NOx, CO, and other pollutants without hurting combustion efficiency [16].

### 1.1 Significance of the study

This research is a significant step towards the development of environmentally friendly, stable emulsified fuels, especially in the context of addressing the pollution problems caused by diesel engines. Emulsified fuel is found to be a potential solution to overcome these emissions and protect the environment from pollution. use of emulsifiers known as surfactants, which keep the emulsion stable for long periods of time. Various studies on emulsified fuel have utilised chemical surfactants; however, biosurfactants are more eco-friendly than chemical surfactants. The novelty of this study is to investigate the potential utilization of biosurfactants for emulsified fuel formation. This research also provides brief details on emulsion type and classification, methods, and ways for the selection of suitable surfactants and biosurfactants for emulsified fuel formations. The objective of this study is to fill out the research gap between theoretical and practical by providing details for the selection of biosurfactants for different emulsified fuel applications. The main object of the study is to provide a potential environmentally friendly solution for emulsified fuel that overcomes emissions effects and protects environments from emissions from diesel engines, making it a unique and essential effort in the field of sustainable energy and transportation solutions.

## 2 Material and methods

**Fig. 2:**
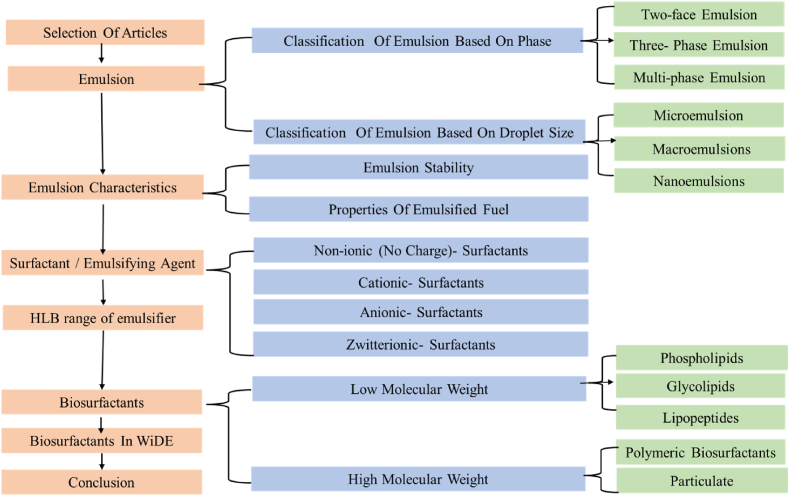
Research Flow chart.

In this study, a literature review is done by searching Researchgate, Scopus, Sciencedirect, and Google Scholar for emulsified fuels and surfactants-related keywords. The primary purpose was to identify articles, research papers, and conference proceedings that examined the synthesis, description, and use of surfactant and biosurfactants in emulsified fuels. The collected material was carefully categorised in chemical surfactants and biosurfactants, so each category underwent a complete review. This study aims to assess the effectiveness of these surfactants in stabilising emulsions, as well as their environmental impact and potential uses in the real world. The study also emphasised the evaluation of various emulsion preparation techniques, investigating their influence on emulsion stability and fuel properties. Furthermore, the physicochemical properties of emulsified fuels, involving parameters such as droplet size distribution, viscosity, and stability, were explored. Putting together the results of this in-depth literature review gave us useful information for the potential use of biosurfactants in emulsified fuels. also highlighted future research directions and addressed challenges for the development of environmentally friendly emulsified fuels for heavy-duty transportation applications. Further selections of materials and methodology are shown in Fig. 2 the research flow chart.

### 2.1 Water-in-oil (w/o) emulsion

**Fig. 3:**
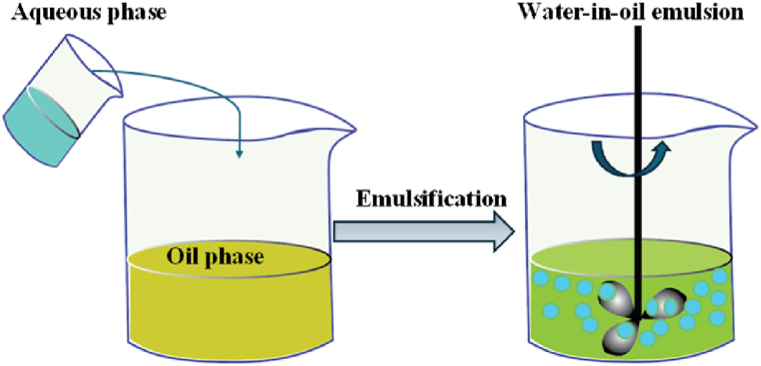
Emulsification process [20].

The term “emulsion” is used to describe a combination of two or more liquids that, owing to liquid-liquid phase separation, are often incompatible with one another. When a drop of one liquid (the dispersed phase) is evenly dispersed across a drop of another, immiscible liquid (the continuous phase), an emulsion is generated [17]. Since interfaces have a very high surface energy and emulsions are often thermodynamically unstable, instability in emulsion occur. For example, fluctuation, Ostwald ripening, coalescence, and phase separation emerge with increasing storage duration [18]. Several kinds of stabilizers, such as emulsifiers, ripening inhibitors, and texture modifiers, have been developed and employed in emulsion compositions to increase emulsion stability [19]. Fig. 3 illustrate the emulsion process.

### 2.2 Classification of emulsions

Emulsions are classified into two main categories based on droplet size and phases.

#### 2.2.1 Emulsion classification based on droplet size

Based on droplet size, emulsions are classified as macroemulsion, microemulsions, and nanoemulsion.

##### 2.2.1.1 Macroemulsion

Macroemulsions having particles size range 1–100 μm are opaque in appearance. These types of emulsion require less amount of emulsifier, and these are thermodynamically unstable because of their tendency for separation and coalescence [[21], [22], [23]].

##### 2.2.1.2 Microemulsions

Microemulsions with particle sizes ranging from 100 to 400 nm are opaque in appearance. These emulsions required a moderate amount of emulsifiers, and these are thermodynamically stable [[21], [22], [23]].

##### 2.2.1.3 Nanoemulsions

Nanoemulsions with particle sizes ranging from 1 to 100 nm are often prepared using gentle methods, emphasizing their small droplet size. They are made by softly blending their ingredients [24]. These are distinct from microemulsions and require small amount of surfactant. These carriers are negatively charged spherical solids with amorphous, lipophilic surfaces [25]. Due to their tiny droplet size, nanoemulsions show high kinetic stability. However, they are thermodynamically unstable, and their stability depends on the specific formulation and ingredients used. To avoid phase separation over time, certain nanoemulsions are designed to be thermally stable [26]. Some examples for commercial use include cosmetics, pharmaceuticals, and agricultural chemicals. Nanoemulsions, in particular, lend themselves to the production of nanomaterials [27]. Based on droplet size emulsions classification detailed in Table 1, Table 2.

**Table 1:** Types of emulsions and their properties [21,[25], [28], [29], [30]].

Emulsion	Macroemulsion	Microemulsion	Nanoemulsions
Type	W/O and O/W	1) biphasic O/W; 2) biphasic W/O; 3) triphasic discontinuous 4) monophasic	W/O and O/W
Droplet Size	1–100 μm	100–400 nm	1–100 nm
Energy Required	Yes	No	Yes
Stability	Kinetic	Thermodynamic	Kinetic

**Table 2:**
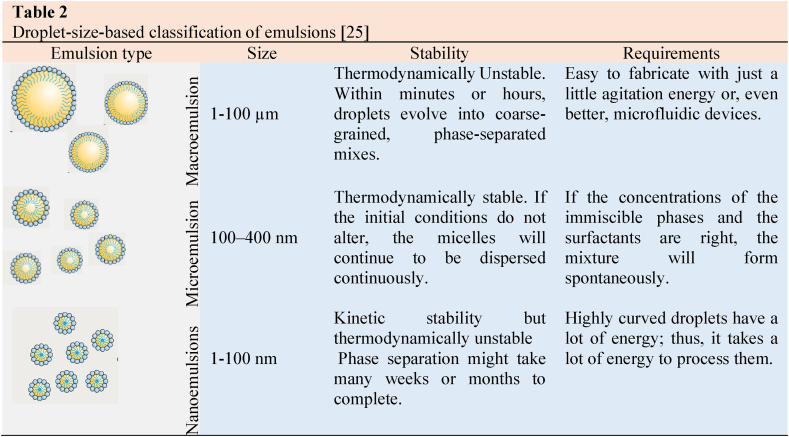
Droplet-size-based classification of emulsions [26]

#### 2.2.2 Emulsion classification based on phases

##### 2.2.2.1 Two-phase emulsion

Both dispersed and continuous phases exist in a two-phase emulsion. It's possible for water and oil to exist in either a continuous or scattered phase. When water acts as the diapered phase and oil as the continuous phase, it refers to the resulting emulsion as Water-in-oil (W/O) emulsion. But when water functions as the continuous phase while oil functions as the scattered phase, it refer to this emulsion as oil-in-water (O/W) emulsion [4]. The fuel's water content affects how water droplets scatter in the solution.

##### 2.2.2.2 Three- phase emulsion

In this emulsion, the intermediate phase, which is a dispersed phase, separates two continuous phases, an interior, and an exterior. Water-in-oil-water emulsion (W/O/W) and oil-in-water-oil (O/W/O) emulsions are two types of three-phase emulsions. Oil is present in the O/W/O emulsion's continuous and inner phases, whereas water makes up the emulsion's scattered phase. A three-phase emulsion's viscosity is above that of a two-phase emulsion [6].

##### 2.2.2.3 Multi-phase emulsion

**Fig. 4:**
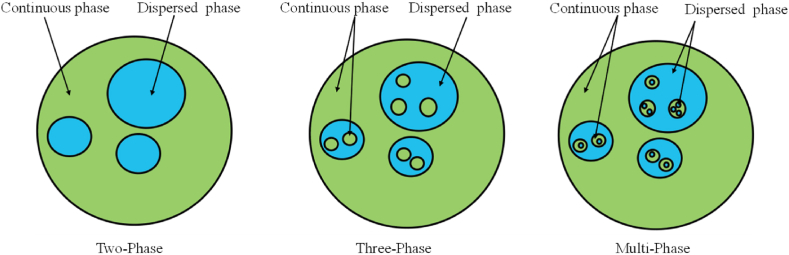
Illustrates the physical structure of different emulsion phases [34,35].

There are two internal phases in these multiphase emulsions, and the droplets of each phase might be the same or different sizes. If the combination is W/O/W or O/W/O, then the emulsion is either multiple or double. While O/W/O emulsions are proposed for use in fuel production, W/O/W emulsions are more often used in the pharmaceutical industry [31]. Several emulsions, such as W/O/W and O/W/O, can be seen in some situations. Common methods for stabilising multiple emulsions include the employment of both hydrophilic and hydrophobic surfactants [32]. The difficulty of multiple emulsions comes from the fact that their little droplets float among much bigger droplets, which spread continuously. The most common kind of emulsion is the W/O/W emulsion, which consists of tiny water droplets suspended in larger oil droplets that are themselves floating in the continuous phase of water, as can be seen in Fig. 4. These emulsions also need the addition of two emulsifiers. The HLB content of one emulsifier should be low, while that of the other should be high [33].

### 2.3 Formation of water-in-diesel emulsion (WIDE)

For the production of emulsion, surface-active agents and a stirrer are used [36]. Emulsion fuels are usually thermodynamically unstable systems, so with time they slowly go back to the two immiscible phases. Mostly, destabilisation of the emulsion fuel process, including creaming, droplet-droplet coalescence, and flocculation [37]. Energy must be provided in order to make emulsions. And the energy must be sufficient to break up one of the phases into small droplets, which can be dispersed and stabilized in the other phase.

**Fig. 5:**
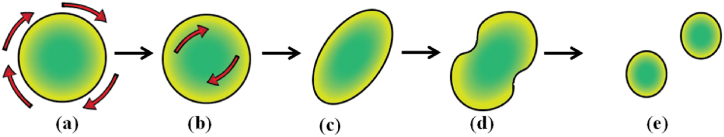
Droplet deformation during emulsification: (a) rotation (b) circulation (c)elongation (d) increase in elongation (e) breaking of droplet [39].

Various types of emulsifying equipment are used for emulsification, such as mechanical mixers, ultrasonic shakers, colloidal mills, and high-pressure conduits [38]. The process of droplet formation and subsequent break-up in emulsions is illustrated in Fig. 5. As shown in Fig. 5(a), emulsions can be created using rotating devices that generate shear forces in the mixture. These shear stresses have various effects on the dispersed droplets. Initially, the droplets begin to rotate, as depicted in Fig. 5(a), which induces momentum within the larger droplets. This momentum causes the liquid inside the droplet to circulate within its boundaries, as shown in Fig. 5(b). As the circulation velocity increases, the droplet begins to elongate, as illustrated in Fig. 5(c). The elongation continues as the droplet stretches further, as seen in Fig. 5(d). Eventually, the momentum causes the droplet to break up. Break-up occurs when the stress from the circulating flow exceeds the stabilising stress due to interfacial tension. Moreover, WIDE forms when water is added to diesel fuel in a way that produces enough shear stress. Under these conditions, the water dispersed within the diesel fuel droplet in the form of tiny droplets.

### 2.4 Stability of emulsion

It is essential that an emulsion maintains its stability over time. It is possible for the engine to shut down if the water from the water-in-diesel emulsion separates before the combustion process. The emulsification technique, the time spent emulsifying, the volume fraction of water in the dispersed phase, the viscosity of the diesel oil in the continuous phase, the stirring speed (or ultrasonic frequency), and the concentration of surfactants are the primary factors that determine the stability of the diesel emulsion [4]. The reason that water and diesel are separate is because their surface tensions are different. To prevent their separation, an emulsifier known as a surfactant is employed to reduce or eliminate differences in surface tension [40]. In order to determine what factors influence the stability of emulsified fuel, the emulsifier dose, oil-to-water ratio, stirring speed, and emulsifying temperature were experimentally analysed. And it was observed that stability was improved by increasing the oil percentage, the stirring rate, and the length of time for the formation of emulsion, but was reduced by increasing the emulsifying temperature [41]. According to studies, W/O emulsion created using an ultrasonic vibrator showed enhanced engine performance and much reduced carbon dioxide emissions than emulsion prepared with mechanical stirring [42]. The development of stable emulsified fuels also relies on the proper surfactant choice, agitator frequency, and agitation time [43]. The surfactant used to emulsify the mixture should not have an adverse effect on the combustion behaviour, such as soot generation, and should be chemically stable [44]. The most utilised surfactants in water in diesel emulsions by researchers are Tweens (polyoxyethylene sorbitan trioleate) and Spans (sorbitan monooleate). The particles get smaller when energy is added to the system, and the emulsion becomes more stable because of this process [45]. The physical properties of oil-water interfacial coatings are influenced by temperature. The surfactant's capacity to dissolve in both the oily and watery phases may also play a role in the emulsion's durability. Additionally, the oily phase of an emulsion's viscosity decreases as the temperature rises. Due to their thermodynamic instability, emulsions constantly change as time passes [46].

### 2.5 Properties of emulsified fuel

Water content, surfactant type and concentration, and emulsification technique all have an impact on the characteristics of water-in-diesel emulsions. Water droplet size, density, kinematic viscosity, and the overall stability of the emulsified fuel are all factors to consider [47].

#### 2.5.1 Viscosity of emulsified fuel

**Fig. 6:**
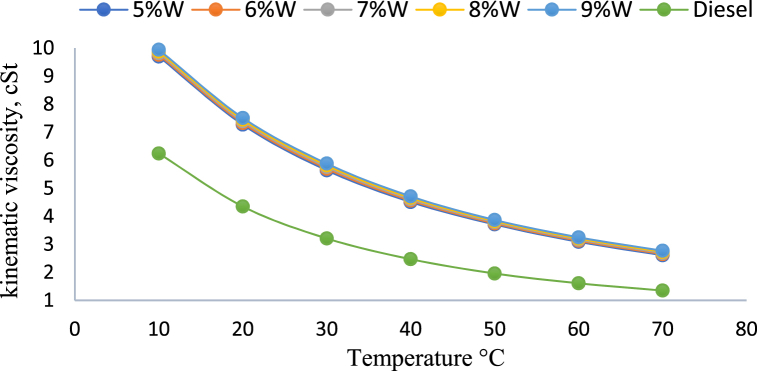
Kinematic viscosity of neat diesel and W/D emulsified fuel at 10–70 °C [52].

**Fig. 7:**
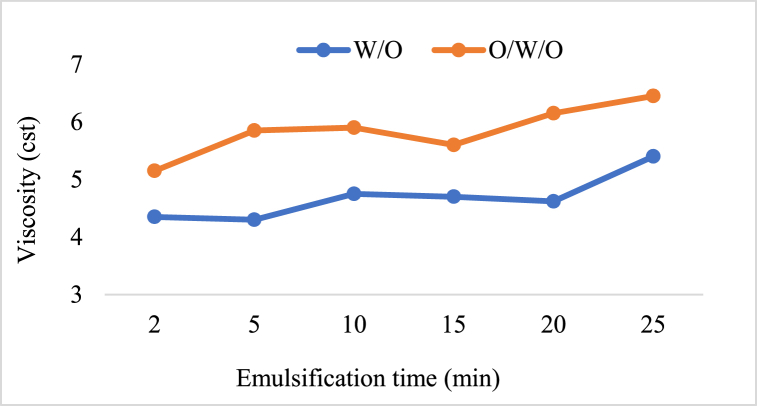
Time-dependent viscosity changes in a 15 percent water-by-volume W/O and O/W/O emulsion [18].

Many variables affect the viscosity impact in W/O emulsions, including droplet size, dispersion shear rate, average droplet size, density, oil viscosity, and temperature [48]. The viscosity of emulsion fuel is higher than that of base fuel [49,50]. Researchers observed that the density and kinematic viscosity of emulsified fuel decreased with increasing temperature, whereas the kinematic viscosity grew with the amount of water utilised in the production of emulsion fuel [51]. As the fuel temperature rose, kinematic viscosity dropped as shown Fig. 6 [52].

(O/W/O) three-phase emulsions have more viscosity than (w/o) two-phase emulsions, while both having the same water content (15 % by volume). It was also discovered that the viscosity increased with the emulsion time as shown in Fig. 7.

#### 2.5.2 Density of emulsified fuel

**Fig. 8:**
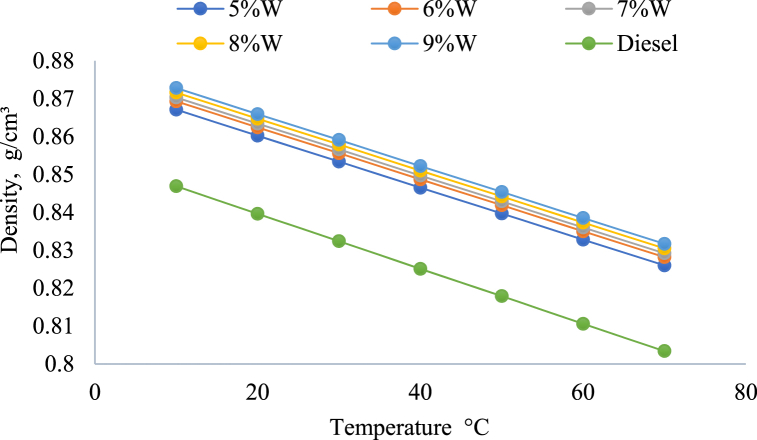
Diesel fuel density of water in diesel emulsion (5–9) wt. percent at 10–70 °C [52].

In general, when the temperature of an emulsion of emulsified fuel rises, its density reduces [51]. According to the results of an experiment, the density of emulsified fuel changes depending on the quantity of water present in the fuel [52]. Overall, the density of emulsified fuel increases with an increase in water content and decreases with an increase in temperature as shown in Fig. 8. The selection of proper parameters and surfactants is crucial for the development of stable and efficient emulsified fuels.

Furthermore, water emulsion fuel's density decreased as its volume rose due to an increase in temperature [51].

#### 2.5.3 Surface tension of emulsified fuel

**Fig. 9:**
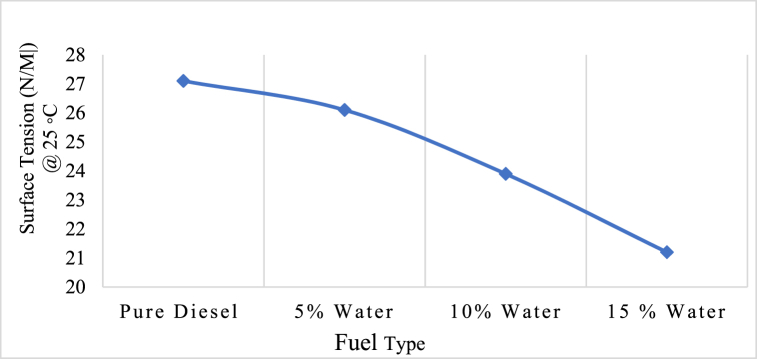
Surface tension of diesel and emulsified fuel [58].

Surface tension is one of the most important physical properties of liquid fuels that affects diesel engine atomization and emulsion stability. Improved atomization and proper air-fuel mixing allow for complete combustion, which in turn increases engine performance and decreases pollutant emissions [[53], [54], [55]]. Additionally, it has been noted that the liquid fuel's high surface tension results in poor atomization and increases the difficulty of droplet formation [56,57]. In one study, surface tension of pure diesel and emulsified fuel with 5 %,10 % and 15 % water concentration was examined and it was found that water concentration reduce the surface tension of emulsified fuel as shown in Fig. 9 [58]. Additionally, several investigations have shown that the surface tension of emulsions decreases as the concentration of surfactants increases [31,[59], [60], [61]].

#### 2.5.4 Calorific value, flash point, and fire point of diesel and emulsified fuel

**Fig. 10:**
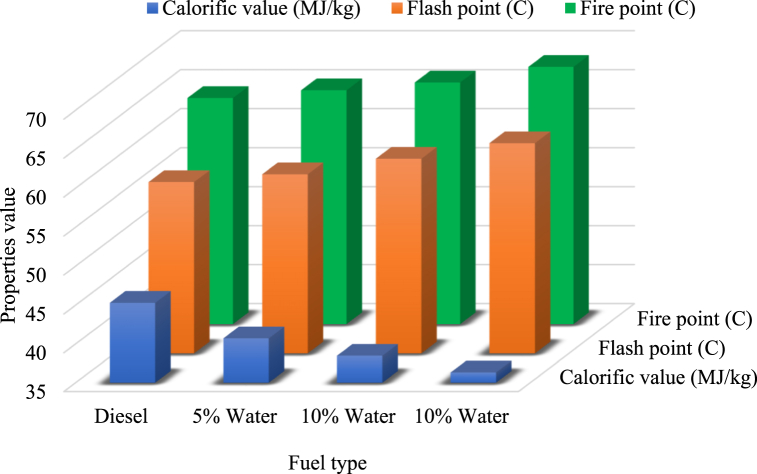
Calorific, flash and fire pint of emulsified fuel [62].

Water concentrations in emulsified diesel fuel effect the properties, N.S. Senthur et al. experimentally found that water concentration changes the calorific value, flash point, and fire point of emulsified fuel. In that study, Pure diesel and three emulsified fuels with 5, 10, and 15 % water concentrations were examined. And it was found that water concentration reduces the calorific value but enhances flash and fire point, as shown in Fig. 10 [62]. It was also reported in different studies that water concentration reduce the calorific value of emulsified fuel [51,63,64] and enhance flash point [65].

### 2.6 Emission and performance characteristics of emulsified fuel

Pollutants such as hydrocarbons (HC), carbon monoxide (CO), and nitrogen oxides (NOx) are released into the environment when diesel engines operate on pure diesel [66]. Fuel modifications with certain amount of water increases brake thermal efficiency (BTE) and decreases emissions of HC, CO, and NOx. Emulsified fuel contributes to secondary atomization and micro-explosions in the combustion chamber because it contains water; pure diesel does not exhibit these characteristics. The micro-explosion reduces the combustion temperature which reduced formation of NOx. Moreover, secondary atomization enhances the rate of the air-fuel mixture, which enhances the efficiency of the engine and reduces the formation of HC and CO. Switching to biodiesel from ordinary diesel is another option to reduce the amount of dangerous pollutants like HC and CO [67]. However, the usage of biodiesel results in an increase in nitrogen oxide emissions (NOx). Water content influences the fuel's viscosity and flashpoint, which in turn impacts the swirling and atomization of the air-fuel mixture. Since various concentrations of emulsion fuel have varied effects on emissions and combustion efficiency due to differences in their physicochemical properties, it is essential to achieve the ideal water concentration for engine performance [68,69]. Overall use of diesel and biodiesel emulsified fuels with appropriate water concentrations helps to overcome emission effects and enhance engine performance.

### 2.7 Research gaps in comparison to previous work

Table 3 describes the various emulsions and emulsifiers applied in different research studies. It was investigated that in various studies for the development of emulsified fuel, chemical surfactants as emulsifiers were utilised. However, limited investigation has been carried out for the utilization of biosurfactants for the development of emulsified fuel. This study highlights a crucial research gap, especially in the range of eco-friendly emulsified fuels. To overcome this research gap, efforts have been made to explore biosurfactants and their potential use in the development of emulsified fuel.

**Table 3:** Illustrates the emulsion type and emulsifiers used in various research studies.

Type	Type	Continues phase. (% vol)	Dispersed phase (% vol)	Emulsifier	Emulsifier Range (% vol)	Reference
Two-Phases	Oil-in- water	Water: (10,20,30,40,50)	Diesel: (90,80,70,60,50)	Sorbitan monooleate (SM)	0.25 to 0.50	[70]
	Water-in-oil	Diesel: (78–86)	Water: (14–22)	NA/(RTES)	NA	[71]
	Water-in-oil	high-speed diesel:(80,85,90,95)	Water: (5,10,15,20)	Sorbitan monolaurate	1	[72]
	Water-in-oil	Diesel: (92)	Water:(5)	(Tween 80 and Span 80)	3	[73]
	Oil-in- water	Water: (10,20)	Diesel: (90,80)	NA/(ultrasonic homogeniser)	N/A	[74]
	Water-in-oil	Diesel:(88,90,93,95)	Water: (2,5,8,10)	Span 80 (Sorbitan monooleate)	2	[75]
	Water-in-oil	Diesel:(92)	Water: (3)	Tween 80 and Span 80	5(1:2)	[76]
	Oil-in- water	Water: (5,10,15)	Diesel (94,89,84)	Tween 80 and Span 80	1(1:1)	[77]
			Biodiesel (94.89,84)			
	Water-in-oil	biodiesel:(89.5,84.5)	Water: (10.15)	Tween 80 and Span 80	0.5 (7: 93)	[78]
	Water-in-oil	Biodiesel (98,96,94)	Water: (2,4,6)	NA	NA	[79]
	Water-in-oil	Diesel + biodiesel (95:5)	Water: (3,5,7)	Tween 80 and Span 80	7(1:2)	[80]
Three-phases	Water-in-oil-in-water	0.1 M NaCl water solution 20	Heavy paraffin oil, Sorbitan monooleate and Aqua deist.,	Hydrophilic emulsifier	1	[81]
	Oil-in-water-in oil	Diesel (ULSD) 78 to 93	Water (5–20)	Tween 80 and Span 80	2	[82]

## 3 Surfactant

Surfactants are emulsifying agents that help stabilise the emulsion. In 1950, the term “surfactant,” short for “surface active agent,” was invented by an Antara product. These substances have at least two parts: one that is lyophobic and insoluble in a given solvent, and another that is lyophilic and miscible with that solvent [83]. This dual nature of the surfactant renders it amphipathic. Hydrophilic and hydrophobic are terms often employed when the solvent is water. The hydrophobic chain is often branching or straight, containing 8 to 18 carbon atoms. The ionicity of the polar head group depends on the overall charge of the molecule. When a surfactant molecule is brought to the surface, hydrogen bonds between water molecules are broken, resulting in lower surface tension. Surfactants lower the surface tension of water from 72 dyne/cm to 35 dyne/cm, making it easier to create an emulsion and spread it over a Wider range of liquids. When surfactants are expressed in dilute proportions, they adsorb onto surfaces [84]. The surfactants are emulsifying agents that enhance the mixing of two immiscible liquids by reducing the surface tension of water.

### 3.1 Charge-based surfactant classification

Surfactants' unique physicochemical properties make them applicable in several domestic and commercial applications [85]. Anionic (negatively charged), cationic (positively charged), nonionic (neutral), and amphoteric/Zwitterionic surfactants are often used to categorise surfactants according to the formal charge present in their hydrophilic heads (which present both positive and negative charges at an intermediate pH). The hydrophilic head's functional type allows for further classification [86].

#### 3.1.1 Non-ionic (no charge)- surfactants

The non-ionic surfactants employed in the production of noisome have no net charge and are relatively non-toxic since they consist of a hydrophilic head group and a hydrophobic tail [16]. The surfactant's hydrophobic part can be alkyl (T), fluoroalkyl, or steroidal. Non-ionic surfactants are frequently employed as emulsifiers, foam-stabilising agents, and wetting agents. They are also well used in several biotechnological processes to make drug carriers more stable and help them dissolve. Ester-linked surfactants, polyoxyethylene alkyl ethers, polyglycerol alkyl ethers, glucosyl dialkyl ethers, crown ethers, polyglycerol alkyl ethers, Brij, Spans (sorbitan esters), and Tweens are all examples of non-ionic surfactants (Polysorbates) [87].

#### 3.1.2 Anionic- surfactants

Anionic surfactants, negatively charged substances, are used in enhanced oil recovery procedures due to their low manufacturing costs, low adsorption, low IFT, and high temperature stability, and in detergent and cleaning industries [18]. Carboxylate, sulphate, sulfonate, and phosphate are the four main types of head polar groups found in anionic surfactants [88].

#### 3.1.3 Cationic-surfactants

Cationic surfactants have positively charged head groups as in the aqueous phase, with nitrogen serving as the major charge carrier [88]. Cationic surfactants, used for antibacterial purposes, have accumulated in the environment, leading to bacterial resistance and high cytotoxicity, making their practical use limited [89].

#### 3.1.4 Zwitterionic- surfactants

**Fig. 11:**
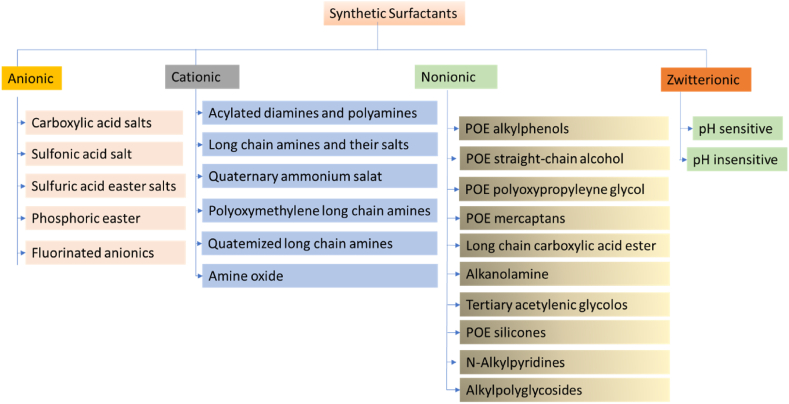
Classification synthetic surfactant [84,92].

Surfactants can be anionic, nonionic, anionic-cationic, or nonionic-cationic zwitterionic, with positive charge associated with ammonium and negative charge often associated with carboxylate [90]. These surfactants are resistant to both heat and salt. However, their exorbitant cost has ceased to be a limitation [91]. A systematic classification of surfactants is given in Fig. 11. The hydrophile-lipophile balance (HLB) value of an emulsifier is a crucial property that determines its ability to form stable emulsions.

### 3.2 Hydrophilic-lipophilic balance (HLB)

The HLB of an emulsifier represents equilibrium between the emulsifier's hydrophilic (water-loving) and lipophilic (oil-loving) groups in terms of size and strength [93]**.** Surfactants have unique HLB numbers indicating their physical properties, determined by the hydrophilic and hydrophobic regions of the molecule, facilitating phase dispersion in an emulsion [94]. HLB is a surfactant metric, calculated by dividing a molecule's hydrophilic-to-hydrophobic ratio, ranging from 1 to 20 as shown in Table 4 with low values for emulsifiers with high lipophilicity and hydrophilicity.

**Table 4:**
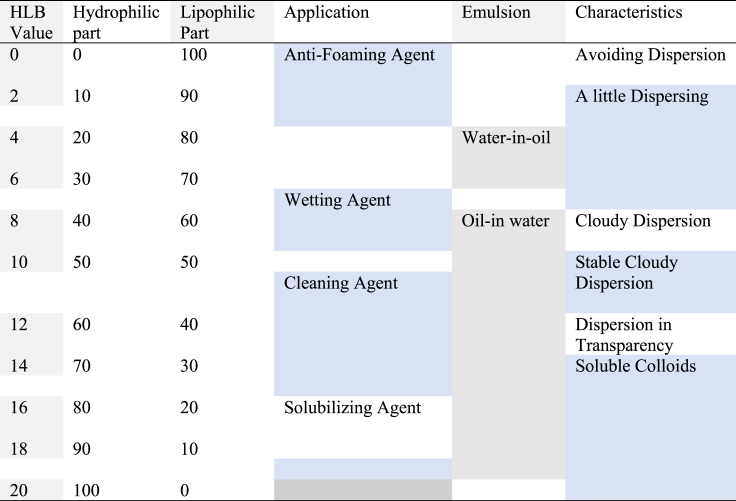
Illustrate the HLB range of emulsifier and its application [95].

Not all hydrophilic and lipophilic compounds can be used as emulsifiers; hydrophilic compounds disperse in water, while lipophilic compounds disperse in oil [74]. The HLB value of a surfactant should match the oil phase's HLB value, but the correlation between hydrophobicity and functioning is not linear. A stable water-diesel emulsion requires a standard HLB value between 4 and 6, as less hydrophilic water disperses in the oil, forming a W/O emulsion. Emulsifying agents with HLB values 8 to 18 are more lipophilic, resulting in an oil-in-water emulsion [75]. An emulsifier's HLB number (high or low) indicates its lipophilic or hydrophilic nature. Using two emulsifiers with varying HLB levels may be more effective for creating stable emulsions, as per research:[96]

(eq.1) $$HLB=\frac{\left(\text{Amount}\;\text{of}\;\text{surfactant}\;1\right)\;\left(\text{HLB}\;\text{surfactant}\;1\right)+\left(\text{Amount}\;\text{of}\;\text{surfactant}\;2\right)\;\left(\text{HLB}\;\text{surfactant}\;2\right)}{\text{Amount}\;\text{of}\;\text{surfactant}\;1+\text{amount}\;\text{of}\;\text{surfactant}\;2}$$

Combining the HLB values of the individual emulsifiers makes it easy to compute the HLB of a combination of two or more emulsifiers; for example, the HLB value of a mixture comprising 60 % TWEEN 80 (HLB = 15) and 40 % SPAN 80 (HLB = 4.3) may be easily calculated [96].

Equation be like this.

**Table:** 

TWEEN 80	60 % X 15.0 = 9.0
SPAN 80	40 % X 4.3 = 1.72
HLB of blend =	10.72

Table 5 provides a comprehensive summary of different emulsifiers, along with their corresponding HLB values and functions. These emulsifiers are specifically classified for their use in water-in-oil emulsions. The HLB values provided are especially appropriate for water-in-oil emulsions. And these emulsifiers are also suggested for creating stable WIDE Fuel.

**Table 5:** Some commonly used emulsifying agent's W/O emulsion their HLB values, characteristics, and functions [97].

Name	HLB value	function
Lecithin	4	w/o emulsion
Sorbitan Oleate	4.3	w/o emulsion
Sorbitan Monostearate NF	4.7	w/o emulsion
Sorbitan Stearate	4.7	w/o emulsion
Sorbitan Isostearate	4.7	w/o emulsion
Steareth-2	4.9	w/o emulsion
Oleth-2	4.9	w/o emulsion
Polyglyceryl-4 oleate	4–6	w/o emulsion
Glyceryl Laurate	5.2	w/o emulsion
Ceteth-2 HLB = 5.3	5.3	w/o emulsion
PEG-30 Dipolyhydroxystearate	5.5	w/o emulsion
Glyceryl Stearate SE	5.8	w/o emulsion
Sorbitan Stearate (and) Sucrose Cocoate	6	w/o emulsion
PEG-4 Dilaurate	6	w/o emulsion

### 3.3 Biosurfactant

Typically, “biosurfactant” refers to surfactants derived from microorganisms [98]. Biosurfactants have an extensive range of applications. Recently, it's been tried as a substitute for chemical-based surfactants in fuel formation emulsions and microemulsions. Because hybrid fuel systems have superior fuel characteristics lower pollutant emissions, they are becoming popular. Rhamnolipid is becoming increasingly popular in fuel preparation, where it is used for glycerol formation in diesel or biodiesel microemulsions, in addition to bio-oil formation in diesel or biodiesel microemulsions [11]. Moreover, since the biosurfactant is a natural emulsifier, the use of a biosurfactant instead of a chemical surfactant in WIDE can improve the quality of emulsified diesel fuel and reduce environmental pollution. Biosurfactants were discovered in the 1960s, but their use has continued into the present day [99,100]. Biosurfactants can be used in several sectors, among them petroleum, pharmaceutical, medical, agricultural, beverage food, textiles, cosmetics, and bioremediation strategies. Besides this biosurfactant, Serratia species are capable of being used as antimicrobial compounds, antitumor compounds, antifouling agents, and emulsifying agents for hydrocarbons [101]. Because of these diverse biological and chemical properties, interest has grown in the use of biosurfactants [102]. Non-ionic, cationic, anionic, and amphoteric surfactants are the four main types of biosurfactants [103]. In comparison to synthetic surface-active compounds, biosurfactants (BS) produced by plants and microorganisms have superior properties, including lower toxicity, biodegradability, low interfacial tensions, and critical micelle concentrations. These are only two examples of the extensive range of unusual physical features shown by these structures. In addition, biosurfactants are gaining popularity in the production of fuel due to their superior performance, low production cost, sustainability characteristics, environmental friendliness, durability at high temperatures and salinity, ability to function across a broad pH range, and a reduced CMC.

#### 3.3.1 Classifications of biosurfactant

Biosurfactants are categorised based on their chemical structure and microbiological source. Biosurfactants typically consist of molecules that combine hydrophilic and hydrophobic properties. Positive, negative, or amphoteric ions often make up hydrophilic molecules, whereas long chains of fatty acids make up hydrophobic ones. The molecular weight, critical micelle concentration, and other characteristics of biosurfactants are often used to categorise them (CMC), the microorganism that produced them, and how they work. Low-molecular-weight biosurfactants are often known to be glycolipids, phospholipids, and lipopeptides, whereas polysaccharides and lipopolysaccharides are the building blocks of high-molecular-weight biosurfactants and a wide range of biopolymers. These are classified in Table 6, which shows their molecular weights (high and low) and types of microbial origin.

**Table 6:** Classification of biosurfactants based on their molecular weight and types of microbial origin [13,27,70,71,75,84,104,[105], [106], [107]].

Group	Molecular weight	Structure		Class
Glycolipids	Low molecular Weight		[108]	Rhamnolipids
				Trehalolipids
				Sophorolipids
phospholipids			[109]	Corynomycolic acid
				Spiculisporic acid
				Phospholipids
				Fatty acids
Lipopeptides			[110]	Surfactin
				Lichenysin
				Peptide lipid
				Wisconsin
				Gramicidin
				Subtilisin
Polymeric biosurfactants	High molecular Weight		[111]	Emulsan
				Alasan
				Biodispersan
				Liposan
				Mannan-lipid protein
				Carbohydrate lipid-protein
Particulate			[112]	Vesicles
				Whole cells

#### 3.3.2 Properties of biosurfactants

Chemical surfactants and emulsifiers have been used in various industries like detergents and soaps, petroleum, textiles, agriculture, medicine, and food. However, since most of these chemicals are hazardous to the environment, having a less- or non-toxic alternative, such as bio-emulsifiers (BE) and biosurfactants (BS), was desirable. Surfactants are amphiphiles, meaning they have both hydrophilic and hydrophobic groups. Because of this property, surfactants may break down surface and interfacial tension and create emulsions. Due to their eco-friendliness and lesser toxicity compared to synthetic surfactants, biosurfactants are gaining popularity as a research subject and for practical use. Surfactants are an exceptionally flexible class of chemicals. There are a number of distinguishing characteristics shared by all surfactants, including their chemical structure, HLB, charge, geographical origin and critical micelle concentration (CMC). Aggregates or micelles may be formed by certain compounds, and these micelles are essential for the compound's unique emulsifying, foaming, dispersing, and detergent-like properties [82]. The CMC, HLB, chemical structure, charge, and origin are only a few of the properties that may be used to classify different types of biosurfactants [113].

##### 3.3.2.1 Critical micelle concentration (CMC)

**Fig. 12:**
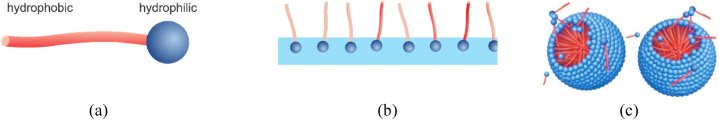
(a) Schematic structure of surfactant (b) Surfactants at an interface (c) Spherical micelles [112].

In aqueous solutions, surfactant molecules only mix to form micelles at a concentration called the crucial micelle concentration [40]. The two main components of surfactants are a polar head group and a non-polar hydrocarbon chain [97], as seen in Fig. 12 (a). It's possible that the molecule's polar region has strong interactions with other polar solvents, such as water. Meanwhile, the non-polar part may form strong interactions with oil and other non-polar solvents. Surfactants, which are composed of two parts, tend to adsorb near interfaces since that's where they can obtain the most favourable energy conditions. Surfactants, for instance, as shown in Fig. 12 (b), they may arrange themselves on a water surface such that the head group is immersed, and the hydrocarbon chain is projecting upwards into the gaseous phase. Since surfactants interact significantly with both phases, they may serve as mediators between them. The interfacial tension thus decreases. The addition of a surfactant significantly enhances the mixing of polar and non-polar phases. Additionally, surfactant molecules self-organize inside a volume phase. Micelles, seen in Fig. 12 (c), are multi-clusters of surfactant molecules whose polar head groups shield their non-polar chains from the surrounding water phase. Micelle production results in a loss of entropy, although this is offset by the elimination of unfavourable interactions between nonpolar surfactant chains and polar liquids. In addition, micelles may take on non-spherical forms like elongated worm-like structures when the temperature and system composition are altered [114].

**Fig. 13:**
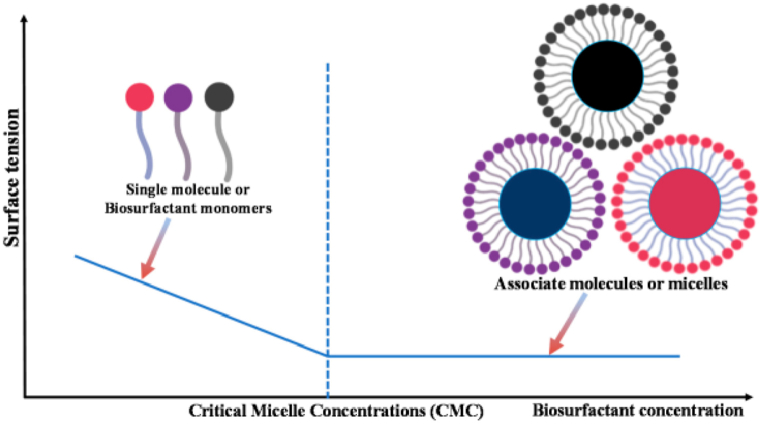
Critical micelle concentration in biosurfactant monomers (CMC) [115].

In aqueous solutions, surfactant molecules start to interact and form micelles at a concentration called the “critical micelle concentration” (CMC). It reduces unfavourable interactions between nonpolar surfactant chains and polar solvents. Since a surfactant's characteristics vary greatly with concentration, the CMC is used as a reference point for research and commercial uses. Fig. 13 illustrates the critical micelle concentration in biosurfactant monomers.

#### 3.3.3 The potential of biosurfactants in WIDE

The potential of biosurfactants in WIDE fuel and diesel engines to create a stable and homogeneous mixture that improves combustion efficiency and reduces emissions [5]. However, the stability of the emulsion can be a challenge, and chemical surfactants are typically used to stabilise the emulsion. Biosurfactants, on the other hand, have been shown to be effective stabilisers of WIDE emulsions [116]. They can also improve the combustion efficiency of the emulsion, leading to lower emissions. One of the advantages of using biosurfactants is their natural origin, which makes them environmentally friendly and biodegradable. They also offer advantages over chemical surfactants, such as lower toxicity, higher biodegradability, and lower environmental impact. Furthermore, biosurfactants are renewable and sustainable, which aligns with the growing demand for more sustainable energy sources. Biosurfactants have been extensively studied as a greener alternative to synthetic surfactants in emulsified fuels. For example, rhamnolipids produced by Pseudomonas aeruginosa have been used for stabilising WIDE, reducing viscosity, and decreasing emissions of particulate matter and hydrocarbon gases [10]. In one study, a test fuel was made with 10 % lemon peel oil (LPO), 10 % water (10W), and 2 % sorbitan monolaurate as a surfactant to make the emulsion stable. In terms of rate of heat release, LPO and LPO10W emulsions performed best, while cetene was used to resolve delayed ignition in LPO10W. The emulsions showed lower opacity, low emissions of air pollutants, and reduced NOx emissions. The renewable and environmentally friendly nature of LPO and LPO10W make them promising alternatives to conventional diesel, contributing to sustainability and green initiatives [110].

Furthermore, biosurfactants also have the potential to improve the lubricity of diesel fuel, reducing wear and tear on engine components and prolonging engine life [116]. They can also reduce the formation of deposits in the engine, which can lead to reduced maintenance costs and improved reliability.

## 4 Conclusion

The diesel engine is a popular source of energy used for sea transportation, industrial sectors, and road vehicles. Studies have been conducted to reduce harmful gas emissions from diesel engines by utilising WIDE fuel as an alternative fuel that may minimise emissions. Water, either by direct injection or viva-emulsified fuel, was found to be effective in the combustion chamber as it reduced peak cylinder temperature. The emulsified fuel separates with the passage of time; therefore, surfactants are used to stabilise the emulsion by reducing the surface tension of diesel and water molecules. Literature suggests that in various emulsified fuel studies, chemical-based surfactants have been traditionally used. However, biosurfactants are more environmentally friendly than traditional chemical surfactants, as they are recommended due to their natural origin and potential to improve emulsified fuel quality. This study aims to highlight the significance of filling research gap by studying in depth a detailed study of emulsion, emulsion categorization, emulsion type, stability of emulsified fuel, and micro-emulsion. In addition, a comprehensive overview of chemical and biosurfactants, their categorization, the selection of surfactants based on their HLB, and their properties is addressed. The cost and availability of biosurfactants is one of the primary obstacles connected with their usage in emulsified fuel systems. Biosurfactant manufacturing is often more costly than chemical surfactant synthesis, and yields are typically lower. However, advancements in biotechnology and microbial fermentation techniques helpful for decreasing costs and enhancing the efficiency of biosurfactant production. Insufficient knowledge of the characteristics and behaviour of biosurfactants in emulsified fuel systems is another obstacle. This research is needed to fully understand the effects of these variables on biosurfactant performance in emulsified fuels. In contrast to previous research, this article provides a unique method for the selection and use of biosurfactants, a kind of natural surfactant that is more ecologically friendly than chemical surfactants, in the production of emulsified fuel. Using emulsified fuels with biosurfactants is recommended as a novel strategy for reducing the emission effect of diesel engines. The potential advantages of emulsified fuels, along with advancements in surfactant technology, provide a favourable alternative for the future of sustainable transportation.

## 5 Future scope of the research

This study aims to scrutinize the emissions from diesel engines and their impact on environmental pollution, specifically concentrating on the application of eco-friendly biosurfactant emulsifiers as alternatives to chemical surfactants in emulsified fuel. Subsequent research will embark on a more extensive exploration of emulsions and emulsified fuels incorporating biosurfactants, with a focus on elucidating their properties and establishing selection criteria based on the hydrophilic-lipophilic balance (HLB) of biosurfactants. Addressing the knowledge gap related to the behaviour of biosurfactants in emulsified fuel systems, the research suggests the use of eco-friendly emulsifiers for water-in-diesel emulsion. The study aims to offer insights into developing a sustainable solution for diesel engines, facilitating the transition to a greener future in transportation by assessing the potential benefits of emulsified fuels utilising biosurfactants.

## Data and code availability

Data will be made available on request.

## CRediT authorship contribution statement

**Asghar Ali:** Methodology, Investigation. **A. Rashid A. Aziz:** Writing – review & editing. **Mhadi A. Ismael:** Writing – review & editing, Writing – original draft, Supervision, Methodology. **Saeed Alqaed:** Supervision, Methodology, Funding acquisition, Data curation.

## Declaration of competing interest

The authors declare that they have no known competing financial interests or personal relationships that could have appeared to influence the work reported in this paper.
